# Patient personas of delayed healthcare-seeking behavior among patients with breast cancer-related lymphedema: A qualitative study

**DOI:** 10.1016/j.apjon.2026.100963

**Published:** 2026-04-22

**Authors:** Ling Wang, Shuyu Yan, Runxi Xiao, Yi Chen, Tingting Cai, Qingmei Huang, Yang Yang, Changrong Yuan

**Affiliations:** a School of Nursing, Fudan University, Shanghai, China; b Department of Oncology, Shanghai Medical College, Fudan University, Shanghai, China

**Keywords:** Breast cancer-related lymphedema, Healthcare-seeking behavior, Delayed care-seeking, Personas, Disease-management, Qualitative study

## Abstract

**Objective:**

This study aimed to explore the heterogeneity of delayed healthcare-seeking behaviors among patients with breast cancer-related lymphedema (BCRL) and to develop patient personas that elucidate distinct barriers and care needs.

**Methods:**

A qualitative descriptive study was conducted using purposive sampling with maximum variation. Patients with BCRL were recruited from a tertiary hospital lymphedema clinic in mainland China between March and May 2025. Semi-structured interviews explored patients’ experiences from symptom onset to health care seeking. Data were analyzed using content analysis with NVivo 20 to generate themes and construct personas based on key dimensions, including patient characteristics, behavioral patterns, and care needs.

**Results:**

Seventeen patients with BCRL participated in the study. Three distinct personas of delayed healthcare-seeking behavior were identified: (1) Unaware–Inattentive, characterized by limited symptom awareness and low health literacy; (2) Avoidant–Denial, characterized by symptom recognition but delayed action due to denial, minimization, or optimistic bias; and (3) Resource-Constrained, characterized by willingness to seek care but impeded by financial, geographic, or healthcare access barriers. These personas demonstrated distinct cognitive, emotional, and behavioral profiles, underscoring the need for tailored intervention strategies.

**Conclusions:**

Delayed healthcare-seeking behavior in patients with BCRL is a heterogeneous and multifactorial process shaped by cognitive, psychological, and structural barriers. Persona-based approaches offer a practical framework for designing targeted nursing interventions to may support long-term self-anagement.

## Introduction

Breast cancer-related lymphedema (BCRL) is one of the most significant and persistent complications among breast cancer survivors, with reported incidence rates ranging from 3% to 8% following sentinel lymph node biopsy to 20% to 30% after axillary lymph node dissection, depending on surgical procedures, surveillance methods, and follow-up duration.^1^, ^2^, ^3^ BCRL arises from damage to lymphatic vessels and nodes secondary to surgery or radiotherapy, leading to impaired lymphatic drainage, progressive tissue changes (e.g., inflammation and fibrosis), and subsequent limb swelling.^4^^,^^5^ As a chronic and progressive condition, BCRL may worsen over time without timely and appropriate management. Although clinical advances in medical knowledge and technology have improved early detection and management strategies, timely healthcare-seeking remains suboptimal.^6^

Nevertheless, most BCRL patients experience delays in seeking health care. Delayed presentation and diagnosis represent a major health care delivery challenge that has a profound impact on patient outcomes and quality of life. Previous studies have consistently reported that lymphedema is substantially underdiagnosed in clinical practice, with only 14% of the cases identified in specialized lymphedema centers, highlighting a significant gap between the true prevalence of the condition and its clinical recognition.^2^ The consequences of delayed intervention are substantial, as early-stage lymphedema can be effectively managed with conservative treatment strategies, whereas advanced-stage lymphedema often requires more intensive interventions that are costly and offer limited therapeutic benefit.^5^^,^^7^ Studies have demonstrated that early intervention and prospective surveillance can significantly improve patient outcomes; however, their implementation remains hindered by persistent practical challenges.^3^^,^^4^ Both systemic and patient-level barriers contribute to delays in diagnosis and healthcare-seeking. Irregular utilization of prospective surveillance programs for early detection remains common, while limited access to screening services and insufficient healthcare resources further restrict the implementation of large-scale screening initiatives.^4^^,^^7^ Age-related factors further exacerbate delays, with older patients facing greater difficulties in accessing timely lymphedema care due to transportation barriers, limited health system navigation support, and competing comorbidities.^4^^,^^8^ In addition to age-related influences, inadequate disease awareness also plays a critical role in delayed healthcare-seeking among patients with BCRL. Due to its underrecognized nature as a chronic condition, patients may underestimate its severity or misinterpret early symptoms as typical postoperative changes. Furthermore, limited access to patient education and suboptimal patient-provider communication can further delay decision-making and initiation of treatment.^9^^,^^10^

Different patient groups may experience distinct barriers contributing to delayed healthcare-seeking. Given the diversity of influencing factors and care needs, it is essential to stratify patients with BCRL into meaningful subgroups and develop corresponding patient personas. Persona-based approaches, together with individualized care models, represent an emerging direction in addressing heterogeneous needs among breast cancer survivors living with or at risk of lymphedema. Modern health care measurement emphasizes patient-centered care, recognizing population heterogeneity in terms of demographic, cultural, and socioeconomic dimensions as critical determinants of health outcomes and care preferences.^11^^,^^12^

Within the framework of precision nursing, classifying patients with delayed healthcare-seeking into distinct personas according to their behavioral, cognitive, and psychosocial characteristics offers a promising strategy to capture heterogeneity in BCRL.^9^^,^^10^ Such personas enable health care providers to identify key determinants of delay, uncover unmet needs, and design mechanism-based, targeted interventions for specific patient subgroups.^13^ Tailoring management strategies according to patient-specific barriers and risk profiles may facilitate earlier detection and intervention, while evidence from prospective surveillance and early intervention programs suggests improvements in lymphedema outcomes and disease control.^14^^,^^15^

Hence, this study aimed to explore the experiences of delayed healthcare-seeking among patients with BCRL, identify key barriers across different patient groups, and develop data-informed personas. These personas are expected to provide important guidance for the development of targeted and effective interventions to promote timely healthcare-seeking and improve clinical outcomes.

## Methods

### Study design and participants

A qualitative descriptive study design was adopted. Participants were purposively recruited from a lymphedema clinic at Jangsu women and children health hospital in mainland China. All participants had completed primary breast cancer treatment (including surgery, chemotherapy, and/or radiotherapy) at the time of recruitment, had a confirmed diagnosis of BCRL, and were receiving lymphedema care. To ensure diversity in patient experiences, a maximum variation sampling strategy was used based on age, treatment history, and educational background.

Inclusion criteria were: (1) diagnosed with unilateral BCRL following breast cancer surgery; (2) aged ≥ 18 years; (3) no evidence of cognitive impairment; (4) provided informed consent. Exclusion criteria were: (1) history of breast cancer recurrence or metastasis; (2) upper limb swelling attributable to other causes (e.g., trauma, allergy); (3) severe cardiac, renal, or other systemic diseases; (4) neurological disorders.

The diagnosis of unilateral BCRL for all participants was confirmed based on clinical medical records from the study hospital. Clinical diagnosis was conducted by professional medical staff, integrating the participants’ breast cancer treatment history and typical lymphedema symptoms, with objective diagnostic methods, including bioelectrical impedance analysis, limb volume measurement, and upper extremity circumference measurement.

BCRL staging was assessed by certified lymphedema therapists at the study hospital according to the International Society of Lymphology (ISL) clinical staging criteria for peripheral lymphedema, and the staging results were extracted from participants’ clinical medical records. The ISL staging criteria for upper extremity BCRL are as follows, Stage 0: no visible edema, with underlying lymphatic dysfunction; Stage I: pitting edema that is reversible with rest/limb elevation, without skin or tissue changes; Stage II (spontaneously irreversible) is characterized by swelling that is no longer effectively reduced by limb elevation. In the early phase, pitting edema may still be present and is accompanied by the onset of tissue fibrosis. As the condition progresses to the late phase, edema becomes predominantly non-pitting, with increasingly evident mild to moderate dermal fibrosis; Stage III: severe irreversible edema with severe dermal fibrosis, skin induration, papillomatosis, and impaired limb function. All participants in this study were classified as Early Stage II, Late Stage II, or Stage III.^16^

### Data collection

First, participants who met the eligibility criteria were apprised of the study's objectives, significance, and the voluntary nature of their participation. To enhance the representativeness of the sample, maximum variation sampling was adopted. Prior to the commencement of the formal interviews, sociodemographic information, including age, gender, marital status, educational level, employment status, disease duration, and self-rated health status, was collected. These data were used to contextualize participants' delayed healthcare-seeking behaviors within their social and clinical backgrounds.

This study adopted a retrospective qualitative design. During the interviews, participants were asked to recall their experiences at the time of symptom onset and their initial healthcare-seeking process, including their treatment status at that time. Delayed healthcare-seeking is defined as an interval of more than three months between symptom onset and the first medical consultation.^17^

In this study, “symptom onset” was determined by participants' self-report of the first occurrence of BCRL-specific symptoms, including subjective swelling/heaviness of the upper extremity and objective visible upper extremity swelling. “First medical consultation” referred to the first time participants sought professional medical advice for BCRL-related symptoms, regardless of whether this occurred in a specialized lymphedema clinic. During the interviews, the timeline of symptom onset and first medical consultation was elicited by anchoring to participants’ current treatment stage, key life events and other salient time nodes. Therefore, the reported delayed duration represents an estimated timeframe rather than an exact measured value.

Semi-structured in-depth interviews were used for data collection. An initial interview guide was developed by the research team and refined through pilot interviews with 2–3 participants. The final guide focused on patients' experiences from initial symptom recognition to diagnosis and treatment. Key questions included: “Could you describe the initial symptoms you experienced? What was your first reaction when these symptoms appeared? How much time passed before you decided to seek medical care? What factors contributed to your decision to delay seeking care.”

Data were collected through face-to-face, in-depth interviews conducted at locations chosen by the participants. Each interview was conducted by two researchers who had clinical experience in oncology nursing and had received formal training in qualitative research interviewing techniques. During the interviews, one researcher served as the primary interviewer, while the other observed, recorded field notes, and documented nonverbal cues and contextual information. Interviews lasted approximately 30–60 minutes. With participants’ consent, all interviews were audio-recorded and transcribed verbatim within 24 hours. Data collection continued until thematic saturation was achieved—that is, when no new or relevant information emerged from additional interviews.

### Data analysis

Data analysis was conducted using qualitative content analysis with the support of NVivo 20 software. Two researchers independently analyzed the transcripts, and discrepancies were resolved through discussion; a third researcher was consulted when consensus could not be reached. The process involved four main steps: (1) Data familiarization and open coding: Transcripts were read repeatedly to gain a holistic understanding. Meaningful statements related to delayed healthcare-seeking were extracted and assigned initial codes. (2) Developing dimensions and categories: Codes were compared, sorted, and grouped into sub-themes and broader categories based on shared characteristics, forming the dimensional framework (descriptions, characteristics, needs) for the patient personas. (3) Persona construction: Patients with similar patterns of characteristics, barriers, and needs were grouped together. Detailed personas were constructed by synthesizing the data within each group, ensuring they accurately represented the shared experiences of that subgroup. (4) Persona description**:** Each persona was developed to capture the heterogeneity of delayed healthcare-seeking behaviors in BCRL, integrating cognitive, emotional, and behavioral dimensions. Descriptions include a narrative summary, key characteristics, and illustrative quotations.

Participants were assigned to one primary persona based on the dominant factor contributing to delayed healthcare-seeking, although multiple factors could coexist. Numerical descriptors were used solely for qualitative contextualization and were not applied as quantitative classification criteria. During analysis, participants’ knowledge of lymphedema was assessed based on their verbal expressions reflecting prior understanding of the condition.

### Rigor

Rigor was ensured through strategies addressing credibility, dependability, confirmability, and transferability. Interviews were conducted by researchers with clinical nursing backgrounds and formal qualitative training, none of whom had prior clinical relationship with participants. Interviews were audio-recorded, transcribed verbatim, and were guided by open-ended semi-structured questions. Credibility was enhanced through iterative, team-based analysis involving multiple researchers, including independent coding of selected transcripts, peer debriefing, and investigator triangulation, with interview data analyzed alongside field notes. Dependability and confirmability were supported through maintaining a detailed audit trail documenting coding process and analytic decisions. Reflexivity was addressed through ongoing team discussions, during which researchers critically reflected on how researchers’ clinical backgrounds and assumptions regarding delayed healthcare-seeking might influence data interpretation. Transferability was facilitated by detailed descriptions of the study context, sampling, participants, and analytic procedures. Member checking was not conducted due to concerns about participant burden during ongoing treatment; instead, credibility was strengthened through prolonged engagement with the data and team-based consensus. The study was reported in accordance with the Consolidated Criteria for Reporting Qualitative Research (COREQ) checklist (Supplementary File 1).

## Results

### Demographic characteristics of participants

Interviews were conducted until data saturation was achieved, resulting in a final sample size of 17 participants. The mean age was 58.0 years (SD = 8.2, range: 36–69). All participants were Chinese women. Most were married (88.2%) and retired (70.6%). Educational levels ranged from secondary education to higher education. The self-reported delay in healthcare-seeking ranged from 4 to 48 months. Most participants were diagnosed with early Stage II lymphedema. Demographic and clinical characteristics are detailed in Table 1.

**Table 1:** General characteristics of the participants (*N*= 17).

ID	Age (years)	Gender	Educational level	Marital status	Employment status	Residence	Accessibility to lymphedema clinic	Health insurance	Financial burden	Lymphedema stage	Lymphedema duration	Delay duration
P1	59	Women	Secondary education	Married	Retired	Urban	Convenient	Medical insurance	Low	Late Stage II	24 months	12 months
P2	58	Women	Secondary education	Divorced	Employed	Urban	Convenient	Medical insurance	Moderate	Early Stage II	34 months	18 months
P3	60	Women	Secondary education	Divorced	Retired	Urban	Inconvenient	Medical insurance	Low	Late Stage II	84 months	43 months
P4	58	Women	Secondary education	Married	Retired	Urban	Convenient	Private insurance	High	Early Stage II	22 months	10 months
P5	56	Women	Higher education	Married	Retired	Urban	Convenient	Medical insurance	Moderate	Early Stage II	11 months	4 months
P6	60	Women	Higher education	Married	Retired	Urban	Convenient	Medical insurance	Low	Early Stage II	33 months	6 months
P7	67	Women	Secondary education	Married	Retired	Urban	Convenient	Medical insurance	Low	Stage III	83 months	40 months
P8	61	Women	Higher education	Married	Retired	Rural	Inconvenient	Medical insurance	Low	Stage III	82 months	48 months
P9	67	Women	Secondary education	Married	Retired	Suburban	Inconvenient	Medical insurance	Low	Early Stage II	26 months	11 months
P10	36	Women	Higher education	Married	Employed	Urban	Convenient	Medical insurance	Moderate	Early Stage II	14 months	7 months
P11	51	Women	Secondary education	Married	Employed	Rural	Inconvenient	NCMS	High	Early Stage II	60 months	6 months
P12	53	Women	Higher education	Married	Employed	Urban	Inconvenient	Medical insurance	High	Early Stage II	21 months	5 months
P13	46	Women	Higher education	Married	Employed	Urban	Convenient	Medical insurance	Low	Early Stage II	23 months	6 months
P14	57	Women	Higher education	Married	Retired	Urban	Convenient	Medical insurance	Low	Early Stage II	72 months	14 months
P15	62	Women	Secondary education	Married	Retired	Urban	Convenient	Private insurance	High	Early Stage II	30 months	16 months
P16	69	Women	Higher education	Married	Retired	Urban	Convenient	Medical insurance	Low	Late Stage II	42 months	20 months
P17	66	Women	Secondary education	Married	Retired	Urban	Convenient	Medical insurance	Low	Late Stage II	58 months	25 months

NCMS, new rural cooperative medical system.

### Personas for delayed health care seeking

Analysis revealed three distinct personas characterizing delayed healthcare-seeking behavior. The detailed description of the characteristics and needs of each type was shown in Table 2.

**Table 2:** Patient personas for delayed health care seeking in BCRL.

Persona Type	Unaware-Inattentive	Avoidant-Denial	Resource-Constrained
Personas			
Individuals	P2, P4, P7, P13, P15, P17	P1, P5, P10, P11, P14	P3, P6, P8, P9, P12, P16
Age(years)	46-67	36-59	53–69
Lymphedema Duration(months)	22-83	11-72	21–84
Lymphedema Stage	Early Stage II, Late Stage II and Stage III	Early Stage II and Late Stage II	Early Stage II, Late Stage II and Stage III
Delay Duration(months)	6–40	4–14	5–48
Education	Secondary education to higher education	Secondary education to higher education	Secondary education to higher education
Description	Patients lack awareness of the problem, do not pay attention to health-related cues, and overlook early signs.	Patients are aware of the issue but deliberately avoid facing it, deny its seriousness, and resist engaging in interventions.	Patients recognize the problem and are willing to take action but face limitations due to social, or structural barriers.
Features	• Lack of lymphedema knowledge • Poor symptom recognition • Unintentional neglect of signs • Inadequate information access	• Adequate lymphedema knowledge • Underestimates severity of lymphedema • Tendency to minimize or rationalize symptoms • Optimistic bias • Passive healthcare-seeking behavior • Emotional burden	• Adequate lymphedema knowledge • Good symptom recognition • Strong intention to seek health care • Barriers to healthcare access • Emotional burden
Intervention Focus/needs	• Health education and symptom recognition training • Follow-up support and symptom monitoring • Access to reliable information and resources	• Risk awareness and motivational intervention. • Cognitive bias correction and risk communication • Targeted health education on disease progression and early treatment • Structured follow-up and symptom monitoring	• Strengthen primary healthcare resources • Remote supervision, telehealth guidance, and online resources • Care coordination with ongoing treatments • Provision of information on available lymphedema services

BCRL, breast cancer-related lymphedema.

Persona 1: The Unaware-Inattentive Type

**Description**: This persona includes individuals who delay seeking medical care primarily due to low health literacy and the inability to recognize early symptoms. The delay is unintentional and results from insufficient health knowledge and limited attention to bodily changes.

**Features:**

• *Lack of lymphedema knowledge*: Patients lack the basic information about lymphedema.
• *Poor symptom recognition*: Patients fail to identify bodily discomfort or abnormal signs of lymphedema.
• *Unintentional neglect of signs*: Their delayed healthcare-seeking behavior stems not from deliberate avoidance, but from insufficient disease knowledge, and poor symptom interpretation skills.
• *Inadequate information access*: The patient is limited in obtaining knowledge about lymphedema.

**Intervention focus/Needs**: These individuals require multicomponent interventions integrating targeted health education, structured follow-up support, and improved access to reliable information. Education should address etiology, early signs, potential complications, and self-monitoring techniques such as limb measurement. Follow-up via clinic visits, phone calls, or digital reminders reinforces timely healthcare-seeking. Accessible materials and guidance to reputable sources, including hospital websites and patient support organizations, ensure ongoing knowledge acquisition.

**Typical patient quote:**

Patient 2 &15: At first, my arm just felt a little uncomfortable, I thought it was a normal reaction and didn't realize it could be lymphedema. No one had ever showed me how to monitor my arm on my own.

Patient 4: When the swelling appeared, I didn't realize my arm swelling was a problem. If someone had reminded me to pay attention to the swelling … I wouldn't have waited so long.

Patient 7 & 13: After my arm became swollen, I did not know it was lymphedema. I was unsure when it was serious enough to seek medical care or where I was supposed to go.

Patient 17: Because I thought lymphedema does not go away easily, I assumed it could not be lymphedema when my swelling subsided the next day. It would really help if someone had explained the early signs of lymphedema to me.

Persona 2: The Avoidant-Denial Type

**Description:** This persona refers to patients who are aware of their symptoms but deliberately postpone medical consultation due to low health awareness, or overly optimistic beliefs that the condition will resolve spontaneously. They tend to dismiss early warning signs, leading to delayed healthcare-seeking and potentially poorer outcomes.

**Features:**

• *Adequate lymphedema knowledge*: Patients understand the disease and are aware of its early symptoms.
• *Underestimates severity of lymphedema*: Patients acknowledge the disease but underestimate its potential impact.
• *Tendency to minimize or rationalize symptoms*: Patients often perceive early symptoms as mild, temporary, or not worth medical attention.
• *Optimistic bias*: Patients believe they are unlikely to experience serious health outcomes.
• *Passive healthcare-seeking behavior*: Patients avoid to seeking health care until symptoms worsen.
• *Emotional burden*: Patients have the feelings of guilt, anxiety, and self-blame due to delay.

**Intervention focus/Needs:** Intervention should focus on psychological counseling of cognitive bias, motivational interviewing, and risk communication. Targeted health education should emphasize the potential progression and consequences of untreated lymphedema, aiming to recalibrate risk perception, correct optimistic bias, and reduce symptom rationalization. Regular follow-up and tailored reminders can further reinforce attention to symptoms and promote timely healthcare-seeking.

**Typical patient quote:**

Patient 1: When I found it swollen at that time, I didn't take it seriously. I didn't think it was a serious problem. If someone had explained it to me in time, I wouldn't have ignored it.

Patient 5 & 14: When my arm was swollen at the beginning, it would go down the next day, so I was not too concerned. It was not until the swelling became more severe that I realized how serious the situation was. Having a system to track my symptoms would have made me feel safer.

Patient 10: Compared with other side effects, this did not seem like a major problem, so I stopped paying attention to it.

Patient 11: I assumed it would resolve on its own, so I ignored it. It would have help if the hospital had contacted me, such as through calls or messages, to check on my arm.

Persona 3: The Resource-Constrained Type

**Description:** This persona includes patients who are motivated to seek care and are aware of their symptoms, but are unable to do so by external structural barriers, such as geographic inaccessibility, limited transportation, inflexible work schedules, conflict medical treatments, or restricted access to health care services.

**Features:**

• *Adequate lymphedema knowledge*: Patients possess sufficient understanding of lymphedema.
• *Good symptom recognition*: Patients can recognize early warning signs.
• *Strong intention to seek* health *care*: Patients are highly motivated to seek health care when symptoms occur.
• *Barriers to healthcare access*: Patients are willing to seek care but are hindered by work obligations, geographic distance, or limited availability of local health care services.
• *Emotional burden*: Patients experience feelings of frustration, helplessness, or resignation.

**Intervention Focus/Needs:** System-level interventions are critical for this group, including strengthening primary care services (especially in rural areas), providing clear information on available lymphedema care, expanding telemedicine to overcome spatial barriers, coordinating with other ongoing treatments to reduce scheduling conflicts, offering flexible clinic hours, and delivering social support services. Ongoing follow-up support and symptom monitoring are also essential to ensure timely healthcare-seeking and improve adherence.

**Typical Patient Quote:**

Patients 3 & 8: The hospital in our region did not know how to manage this condition. It would have been great to have some reliable information online, and to receive guidance from a health care professional, even remotely.

Patient 9: My home is in a remote area, and it is too far to travel to the city. There is no one at home to accompany me. I would prefer a nearby clinic where I can receive appropriate lymphedema care.

Patient 6 &12: At that time, when my arm was swollen, I was unsure where to seek treatment for the edema and this delay caused some inconvenience. I would have felt better if I had known which clinics to go to and how to get there.

Patient 16: I knew immediately that it was lymphedema, but there was nothing I could do about it. I was undergoing chemotherapy at the time and couldn't address the issue. It would have helped if the hospital could coordinate my cancer treatment and lymphedema care.

### Discussion

The results of this research identify that delayed healthcare-seeking behavior in BCRL is not a homogenous phenomenon but a heterogenous process influenced by patients’ perception-oriented cognition, emotional response, and environmental constraints. This is congruent with new evidence that healthcare-seeking heterogeneity demands differentiated comprehension and individualized interventions.^18^^,^^19^ The identification of three personas— the Unaware–Inattentive Type, Avoidant–Denial Type, and Resource–Constrained Type—provides a conceptual framework for understanding the multifaceted nature of health care delay beyond a one-size-fits-all perspective.

### Main findings

The Unaware-Inattentive Type represents patients who experience delays in healthcare-seeking primarily due to insufficient knowledge and limited health literacy. This type often fails to recognize early symptoms of lymphedema, frequently attributing initial swelling or discomfort to normal postoperative changes or other benign causes. This finding is consistent with recent evidence. Recent studies have demonstrated that insufficient knowledge of lymphedema is associated with delayed preventive behaviors and suboptimal self-management.^20^^,^^21^ Buki et al. found that Latina breast cancer survivors had never heard of lymphedema, highlighting persistent informational gaps even within established health care systems.^22^ Delayed care in this type often reflects systemic gaps in patient education and symptom surveillance rather than patient neglect. Research by Tse et al. shows that early surveillance and intervention programs can help bridge knowledge gaps and facilitate earlier symptom recognition.^4^ Developing models to find high-risk patients in this group could help provide education and monitoring early.

The Avoidant-Denial Type includes patients who recognize symptoms but postpone seeking care. These patients are aware of disease-related signs. However, they often choose to ignore or downplay them, leading to delayed healthcare-seeking behavior. Patients in this group usually recognize physical changes or discomfort. They often interpret these signs as harmless or temporary. Unlike patients with limited lymphedema-related knowledge and poor symptom awareness or limited access, they generally have basic knowledge about their condition. They also do not face major financial or logistical barriers to care. Their delay comes mainly from cognitive and emotional factors. These include denial, minimizing the threat, and strong optimism bias. Such delays can lead to missed chances for early treatment and poor outcomes.^23^ These patterns are consistent with the Health Belief Model, particularly the constructs of low perceived severity and low perceived susceptibility, which are known to reduce engagement in preventive and healthcare-seeking behaviors.^24^ Intervention strategies should therefore target the specific cognitive and emotional barriers observed in this patient group. Risk communication should be tailored to emphasize the potential severity and long-term consequences of symptom neglect, using personalized messaging and real-life patient narratives to increase perceived threat. Research suggests that peer support programs, cognitive-behavioral interventions, and culturally adapted educational materials can effectively reduce psychological barriers to healthcare-seeking.^25^^,^^26^ Motivational interviewing techniques can be employed in clinical and community settings to explore ambivalence, enhance intrinsic motivation, and support behavior change. Digital health tools may help reinforce the importance of timely care and prompt reevaluation of symptoms that persist.^27^^,^^28^ The integration of mental health support within lymphedema care pathways may be particularly crucial for this persona.

The Resource-Constrained Type represents patients who are motivated to seek care but encounter external structural barriers that impede timely access to appropriate services. These barriers include geographic inaccessibility, limited healthcare resources, and competing work responsibilities, which are consistent with existing evidence highlighting the impact of socioeconomic and health system factors on cancer survivorship.^8^ Socioeconomic disadvantage, including unemployment and financial constraints, as well as geographic barriers such as rural residence, have been consistently associated with delayed cancer care and prolonged healthcare-seeking intervals.^8^ Although patients in this group are willing to engage in health care, they often lack the structural support necessary to act on this intention. Work demands and limited medical resources make it hard to prioritize health care, resulting in delayed care even when symptoms are recognized. Helping the Resource-Constrained Type requires system-level changes to make health care easier to reach and use. Personalized care approaches that integrate individual clinical, psychosocial, and contextual factors, including geographic and social support considerations, have been increasingly recognized as essential for optimizing BCRL management.^8^^,^^12^ Programs such as telemedicine, community-based care, and financial assistance may be especially helpful for these patients. In this study, some participants reported that lymphedema symptoms emerged during chemotherapy, when management was often deferred due to competing treatment priorities, leading to delays in care. This finding suggests the need to better integrate lymphedema management into ongoing cancer treatment pathways to improve care continuity.

In real-world clinical settings, patients may experience multiple, coexisting factors contributing to delayed healthcare-seeking, such as limited acknowledgement of lymphedema alongside restricted access to healthcare resources. Accordingly, the personas identified in this study should be interpreted as dominant patterns rather than exhaustive or mutually exclusive categories. From a clinical perspective, health care providers can pragmatically distinguish between personas by identifying the primary and secondary drivers of delay, prioritizing interventions based on the most salient barrier while simultaneously addressing coexisting challenges. In this way, the persona framework functions as a heuristic tool to support coordinated, patient-centered nursing interventions that accommodate the complexity of individual healthcare-seeking experiences.

### Implications for nursing practice and research

Identifying distinct patient personas has important implications for precision nursing in BCRL care. Standard approaches that give the same education or support to all patients may not meet their different needs. A persona-based approach shows that interventions should target the specific factors that cause delayed healthcare-seeking in each group. Developing persona-specific interventions means understanding the main barriers and supports for each type. For the Unaware-Inattentive Type, interventions should prioritize improving disease knowledge, enhancing early symptom recognition, and implementing proactive surveillance strategies. For the Avoidant-Denial Type, interventions should raise risk awareness with personalized education and address cognitive biases through motivational interviewing. For the Resource-Constrained Type, interventions should remove structural barriers, including facilitating health care navigation, providing financial support, and improving access to care services.

### Limitations

The persona-based framework offers useful insights into delayed healthcare-seeking behavior among patients with BCRL. Several limitations should be considered when interpreting the findings. First, this study recruited participants from a single lymphedema clinic, which may have introduced selection bias. Consequently, the personas identified in this study may not fully capture the full spectrum of healthcare-seeking behaviors among all individuals with BCRL, and the transferability of the findings may be limited. Future studies recruiting from surgical or oncology settings could better capture the full spectrum of healthcare-seeking behaviors. Second, the timeline of BCRL symptom onset and healthcare-seeking was based on participants’ self-report data, which may introduce recall bias and lead to blurred time points of the delay duration. The delay duration reported in this study is an estimated approximate value, which may not fully reflect the actual accurate delay situation of the participants. Future research should test these personas in different populations and health care systems. It should develop standard tools to identify personas and evaluate persona-specific interventions. Long-term studies could examine how patients move between personas over time. This would provide a better understanding of the changing patterns of help-seeking. Developing and testing nursing interventions tailored to each persona is an important next step for improving BCRL care outcomes.

## Conclusions

Identifying distinct patient personas in delayed healthcare-seeking for BCRL helps us understand the complexity of healthcare-seeking behaviors in cancer survivorship. The Unaware-Inattentive Type, Avoidant-Denial Type, and Resource-Constrained Type each exhibit unique barriers and require tailored intervention strategies. These interventions should target their specific barriers and build on their strengths. Applying persona-based understanding in clinical practice and policy can may help inform strategies to promote earlier detection, timely care, and long-term self-management support. This precision nursing approach recognizes the variety of patient experiences and provides a practical framework for delivering personalized, effective care.

## CRediT authorship contribution statement

**Ling Wang**: Conceptualization, Methodology, Formal analysis, Writing - Original draft. **Shuyu Yan**: Conceptualization, Methodology, Formal analysis, Writing - Original draft. **Runxi Xiao**: Methodology, Data curation, Investigation, Writing - Original draft. **Yi Chen**: Conceptualization, Methodology, Data curation, Investigation. **Tingting Cai**: Writing - review and editing, Methodology, Software. **Qingmei Huang**: Visualization, Resources, Methodology, Data curation, Software. **Yang Yang**: Conceptualization, Methodology, Data curation, Software. **Changrong Yuan**: Conceptualization, Methodology, Writing - review and editing, Resources, Funding acquisition. All authors read and approved the final manuscript. Ling Wang and Shuyu Yan contributed equally to this work.

## Ethics statement

This study was approved by the Institutional Review Board of the School of Nursing, Fudan University (IRB No. 2024–4-12) and was conducted in accordance with the 1964 Helsinki Declaration and its later amendments or comparable ethical standards. All participants provided written informed consent.

## Data availability statement

The data that support the findings of this study are available from the corresponding author upon reasonable request. The data are not publicly available due to privacy and ethical restrictions.

## Declaration of generative AI and AI-assisted technologies in the writing process

ChatGPT was used in the preparation of this manuscript to improve clarity, grammar, and readability. All scientific content, study design, data analysis, interpretation of results, and conclusions were performed solely by the authors.

## Funding

This work was supported by the National Natural Science Foundation of China (Grant No. 72374048, 2023) and the Nursing Research Fund Project of School of Nursing, Fudan University (Grant No. FNSF202403). The funders had no role in considering the study design or in the collection, analysis, interpretation of data, writing of the report, or decision to submit the article for publication.

## Declaration of competing interest

The authors declare that there is no conflict of interest. The author, Dr. Tingting Cai, is an editorial board member of *Asia–Pacific Journal of Oncology Nursing*. The article was subject to the journal's standard procedures, with peer review handled independently of Dr. Cai and the research groups.
