# Supplementary material for: Patient personas of delayed healthcare-seeking behavior among patients with breast cancer-related lymphedema: A qualitative study
Source: Asia Pac J Oncol Nurs. 2026 Apr 22;13:100963. doi: 10.1016/j.apjon.2026.100963 (PMC13214556; doi:10.1016/j.apjon.2026.100963)
Supplement: Multimedia component 1 [file mmc1.docx]

**Consolidated Criteria for Reporting Qualitative Research (COREQ): 32-item checklist**

Please indicate where each item is reported in the manuscript.

| No. | Item | Guide questions / description | Section |
| --- | --- | --- | --- |
| 1 | Interviewer/facilitator | Which author(s) conducted the interview or focus group? | Methods: Data Collection |
| 2 | Credentials | What were the researcher’s credentials? (e.g. PhD, MD) | Methods: Data Collection |
| 3 | Occupation | What was their occupation at the time of the study? | Methods: Data Collection |
| 4 | Gender | Was the researcher male or female? | Methods: Data Collection |
| 5 | Experience and training | What experience or training did the researcher have? | Methods: Data Collection |
| 6 | Relationship established | Was a relationship established prior to study commencement? | Methods: Rigor |
| 7 | Participant knowledge of the interviewer | What did the participants know about the researcher? | Methods: Rigor |
| 8 | Interviewer characteristics | What characteristics were reported about the interviewer/facilitator? | Methods: Rigor |
| 9 | Methodological orientation and theory | What methodological orientation underpinned the study? | Methods: Study Design |
| 10 | Sampling | How were participants selected? | Methods: Study Design |
| 11 | Method of approach | How were participants approached? | Methods: Study Design |
| 12 | Sample size | How many participants were in the study? | Results: Demographics |
| 13 | Non-participation | How many people refused or dropped out? Reasons? | Methods: Data Collection |
| 14 | Setting of data collection | Where was the data collected? | Results: Demographics |
| 15 | Presence of non-participants | Was anyone else present besides participants and researchers? | Methods: Data Collection |
| 16 | Description of sample | What are the important characteristics of the sample? | Results: Demographics |
| 17 | Interview guide | Were questions or guides provided? Was it pilot tested? | Methods: Data Collection |
| 18 | Repeat interviews | Were repeat interviews carried out? | Methods: Study Design |
| 19 | Audio/visual recording | Did the research use audio or visual recording? | Methods: Data Collection |
| 20 | Field notes | Were field notes made during or after interviews? | Methods: Data Collection |
| 21 | Duration | What was the duration of the interviews? | Methods: Data Collection |
| 22 | Data saturation | Was data saturation discussed? | Methods: Data Collection |
| 23 | Transcripts returned | Were transcripts returned to participants for comment? | Methods: Study Design |
| 24 | Number of data coders | How many data coders coded the data? | Methods: Data Analysis |
| 25 | Description of the coding tree | Was a description of the coding tree provided? | Methods: Data Analysis |
| 26 | Derivation of themes | Were themes identified in advance or derived from the data? | Methods: Data Analysis |
| 27 | Software | What software was used to manage the data? | Methods: Data Analysis |
| 28 | Participant checking | Did participants provide feedback on the findings? | Methods: Rigor |
| 29 | Quotations presented | Were participant quotations presented and identified? | Results |
| 30 | Data and findings consistent | Was there consistency between data and findings? | Results |
| 31 | Clarity of major themes | Were major themes clearly presented? | Results |
| 32 | Clarity of minor themes | Was there a description of minor themes or diverse cases? | Results |
